# How adherence to the updated physical activity guidelines should be assessed with accelerometer?

**DOI:** 10.1093/eurpub/ckac078

**Published:** 2022-08-26

**Authors:** Henri Vähä-Ypyä, Harri Sievänen, Pauliina Husu, Kari Tokola, Ari Mänttäri, Olli J Heinonen, Jarmo Heiskanen, Kaisu M Kaikkonen, Kai Savonen, Sami Kokko, Tommi Vasankari

**Affiliations:** The UKK-Institute for Health Promotion Research, Tampere, Finland; The UKK-Institute for Health Promotion Research, Tampere, Finland; The UKK-Institute for Health Promotion Research, Tampere, Finland; The UKK-Institute for Health Promotion Research, Tampere, Finland; The UKK-Institute for Health Promotion Research, Tampere, Finland; University of Turku, Turku, Finland; LIKES Research Centre for Physical Activity and Health, Jyväskylä, Finland; Oulu Deaconess Institute Foundation, Oulu, Finland; Kuopio Research Institute of Exercise Medicine, Kuopio, Finland; Kuopio University Hospital, Kuopio, Finland; University of Jyväskylä, Jyväskylä, Finland; The UKK-Institute for Health Promotion Research, Tampere, Finland; Faculty of Medicine and Health Technology, Military medicine, Tampere University, Tampere, Finland

## Abstract

**Background:**

The aerobic part of the recently updated physical activity (PA) guidelines for adults recommends at least 150 min of moderate or at least 75 of vigorous-intensity PA or an equivalent combination of both. PA can be accumulated of any bout duration. On an absolute scale, moderate-intensity threshold is 3 metabolic equivalents (METs) and vigorous 6 METs. On a scale relative to individual’s personal capacity, moderate-intensity threshold is 40% and vigorous 60% of the oxygen uptake reserve. In this study, the adherence to the new guidelines was evaluated using both absolute and relative thresholds.

**Methods:**

Totally, 1645 adults aged 20–64 years, participated in this population-based study and their cardiorespiratory fitness (CRF) was estimated with 6-min walking test. The participants with estimated maximal oxygen uptake <7.9 MET were categorized as low CRF group and the others as adequate CRF group. The participants were instructed to wear a triaxial hip-worn accelerometer for 1 week and their adherence to PA guidelines was assessed from the accelerometer data.

**Results:**

The adequate CRF group had higher adherence to PA guidelines with the absolute thresholds, but the use of relative thresholds inverted the results. The adherence varied from 20% to 99% in the total sample depending on the analysis parameters of accelerometer data.

**Conclusions:**

The absolute thresholds provide a more appropriate basis to assess the adherence to PA guidelines in population-based samples and interventions. The use of individually determined relative thresholds may be more useful for individual exercise prescriptions in PA counseling.

## Introduction

Regular physical activity (PA) and high cardiorespiratory fitness (CRF) have numerous scientifically documented health benefits.^1^^,^^2^ The aerobic part of the recently updated PA guidelines for adults recommends at least 150–300 min of moderate PA (MPA) or 75–150 min of vigorous PA (VPA) weekly, or some combination of them.^3^^,^^4^ A striking change from the previous PA guidelines is that the at least 10-min bouts are no more required for the accumulation of relevant PA. Recent studies employing device-measured PA have indicated that the total volume of moderate-to-vigorous PA (MVPA) is related to many health benefits, whereas time-specific bouts are not essential.^2^^,^^5^

PA-related health benefits and self-reported MVPA have shown an inverse, curvilinear dose–response relationship.^2^ Even small amounts of PA confer health benefits while they are most evident for the least active individuals.^6^ The health benefits continue across the full range of commonly achievable volumes, although they have diminishing returns for MVPA levels over 150–300 min per week.^2^ However, it seems that daily 30–40 min of accelerometer-measured MVPA may attenuate the association between sedentary time and risk of death,^7^ being substantially lower than previously estimated 60–75 min based on self-reported data.^8^

High CRF is associated with a significant reduction in all-cause mortality at any level of habitual PA, without evidence of a plateau effect or U-shaped association.^1^ CRF is determined by maximal oxygen uptake (VO_2_max) during exercise. CRF is more strongly associated with all-cause mortality than self-reported PA in men and women.^9^ The minimum CRF conferring substantial risk reduction is estimated to be 7.9 MET (metabolic equivalents, 1 MET = 3.5 ml/kg/min of oxygen consumption).^10^

To assess trends regarding the adherence to PA guidelines, it is important to regularly measure population-level PA with valid methods. Such methods should be able to measure the frequency, duration and intensity of PA, and desirably also the type of activity and its context. Positive effects of PA on health and fitness are associated with the intensity, duration and frequency of physical effort.^9^ The measurement methods can be divided into self-reports and device-based.^11^ The self-reports have low respondent burden and cost but have problems in ascertaining the frequency, duration and intensity of PA, capturing all domains of PA especially light PA, short bouts and sporadic movements, social desirability bias and the cognitive demands of recall.^11^^,^^12^ The device-based methods can assess PA in a more standardized manner regardless of the current fitness level and body weight which both may influence the subjectively perceived and reported intensity of PA.^13–15^ However, there are no guidelines or recommendations how to assess the adherence to meeting the guidelines of PA with device-based methods. The device-based methods are unable to measure all activities equally well and cannot measure effect of carrying or lifting heavy loads or weights.^11^ Thus, they are not able to measure the muscle strengthening part of the guidelines.^16^ Self-reported data can supplement device-based data, for example, by providing information on the specific type or context of PA.^17^^,^^18^

The intensity of aerobic PA can be expressed in either absolute or relative terms.^4^ Absolute intensity is the amount of energy expended during the given activity without considering a person’s CRF or aerobic capacity. Moderate-intensity activities have a MET value of 3–5.9 METs and vigorous-intensity activities have a MET value of 6 or greater.^4^ In contrast to absolute intensity, relative intensity denotes the level of effort relative to a person’s individual maximum aerobic capacity.^4^^,^^19^ The relative intensity can be estimated using the percentage of oxygen uptake reserve (VO_2_R), VO_2_max, heart rate (HR) reserve or maximum HR.^19^ The use of relative intensity has been recommended when it is feasible in device-based PA studies, but in large-scale population studies, it can be too laborious and costly to conduct individual exercise testing in laboratory conditions.^7^^,^^20^ However, the 6-min walk test (6MWT), a cost-effective and well-documented field test of CRF,^21^^,^^22^ has recently been validated for predicting VO_2_max also among healthy adults.^23^

It is known that the choice of parameters employed in the analysis of device-measured PA data can substantially affect the results. The use of relative intensity thresholds may lead to paradoxical results regarding the total amount of MVPA time.^24^ Likewise, the use of different epoch lengths and cut-points to define the intensity (MPA or VPA) can essentially change the estimates of the accumulated PA time.^24^ Shorter epoch lengths will capture instantaneous and sporadic instances of movement, which are most likely missed with longer epochs due to the inherent smoothing effect. On the other hand, the smoothing effect of the long epoch allows the intensity temporarily to drop below the cut-point.^25^^,^^26^ The cut-points together with the epoch length determine the time spent in MPA and VPA levels. The selected intensity cut-points should be validated in a sample population closely matching the study group of interest and the selected epoch length should be the same that was used to validate the cut-points.^27^

The purpose of the present study is to systematically examine the device-based adherence to the aerobic part of the updated 24-h movement guideline in Finland using both absolute and relative scale. The new guideline for adults aged 18–64 years combines recommendations for the PA, sedentary behavior and sleep across the whole day (Supplementary figure S1). Also, a scheme for assessing population-based adherence to the aerobic part of the PA guidelines is outlined.

## Methods

This study is based on a subsample of the population-based FinFit2017 study.^28^ The sample comprised of 1645 participants (658 men, 987 women), aged 20–64 years, who completed 6MWT and had 24-h daily wear time of the accelerometer at least for 4 days during seven consecutive days. Potential participants for the FinFit2017 study were drawn from the Population Information System by the Finnish Digital and Population Data Services Agency. The sample was collected in seven city-centered regions of Finland: 300 men and women from both Helsinki and Tampere regions and 150 men and women from each of Turku, Kuopio, Jyväskylä, Oulu and Rovaniemi regions spread across five age groups (20–29, 30–39, 40–49, 50–59 and 60–69 years). Other inclusion or exclusion criteria were not used and participation in the study was voluntary. Health and fitness examination were conducted at the local research centers in the above-mentioned cities. Before the 6MWT was conducted a health screening^23^ to exclude participants with health limitations restricting their ability to walk. The data collection was conducted from autumn 2017 to spring 2019.

The coordinating ethics committee of the Regional Ethics Committee of the Expert Responsibility area of Tampere University Hospital gave the ethical approval for the study (R17030). All participants gave signed informed consent before participation. All methods were performed in accordance with relevant guidelines and regulations and all research was performed in accordance with the Declaration of Helsinki.

The CRF was estimated using the 6MWT, where the participants were asked to walk back and forth along the 15-m walking track as fast as possible for 6 min.^23^ The HR was recorded with an HR monitor (Polar M61, Polar Electro, Kempele, Finland). For men, the VO_2_max (ml/kg/min) was predicted by the walking distance in 6 min (6MWD), age, body mass index (BMI), body height and HR at the end of the test. For women, the prediction was based on the 6MWD, body weight, and age. The standard error of the estimate is 3.60 ml/kg/min for men and 3.51 ml/kg/min for women.^23^ The prediction equation for men is following:

$$\begin{array}{c} {\mathrm{VO}}_{2}m\mathrm{ax}=110.546+0.063\times \left(6M\mathrm{WD}\right)-0.250\times \left(\mathrm{age}\right)\\ -0.486\times \left(\mathrm{BMI}\right)-0.420\times \left(\mathrm{height}\right)-0.109\times (\mathrm{HR}) \end{array}$$

The prediction equation for women is following:

$$\begin{array}{c} {\mathrm{VO}}_{2}m\mathrm{ax}=22.506-0.250\times \left(\mathrm{weight}\right)+0.051\times \left(6M\mathrm{WD}\right)\\ -0.065\times \left(\mathrm{age}\right) \end{array}$$

According to predicted VO_2_max values, the participants were divided into low and adequate CRF groups. The low CRF group comprised persons, whose estimated VO_2_max values were <7.9 METs (27.65 ml/kg/min), whereas the adequate CRF group comprised persons with estimated VO_2_max values of at least 7.9 METs.^10^

A triaxial accelerometer (UKK RM42, UKK Terveyspalvelut Oy, Tampere, Finland) was used to measure participants’ PA. The device was attached to a flexible belt with an instruction to wear the belt so that the accelerometer was on the right hip during waking hours and on the non-dominant wrist during bedtime for seven consecutive days, except during shower and other water activities. The acceleration signal was collected at 100 Hz sampling frequency, ±16 g acceleration range, and 0.004 g resolution. After the 1-week measurement, the accelerometers were returned, and the raw data were stored on a hard disk for further analysis.

The raw accelerometer data was analyzed in 6 s epochs according to our standard procedure.^29–31^ For each epoch, mean amplitude deviation values of the resultant acceleration signal,^29^^,^^30^ and of the acceleration signal in each orthogonal direction^31^ were calculated. The epoch-wise acceleration values were converted to METs. The accuracy of the MET-estimation is about 1.2 MET for bipedal locomotion over a wide range of speed.^30^ The MET values of the 6 s epochs were further smoothed by filtering the data with 1 min and 6 min exponential moving averages (EMAs).^24^ Presumably, the 6 s epochs capture all relevant bodily movements produced by skeletal muscles, whereas the longer 1 min and 6 min EMA simulate the metabolic responses to PA.^32^^,^^33^ The time course of the physiological responses is wide depending on the intensity and duration of the activity as well as the training status and fitness of the person.

The intensity of PA at each epoch time point was classified into combined MVPA and VPA on an absolute scale and on a scale relative to individual’s personal capacity. The absolute cut-points were the standard 3.0 MET and 6.0 MET and the relative cut-points 40% and 60% of the VO_2_R.^19^ The days containing non-wear time were excluded from the analysis.^29^ The mean daily guideline PA time was calculated as the sum of MPA time and doubled VPA time. The weekly time was obtained by multiplying the mean daily time by seven. The participant was classified as sufficiently physically active if the weekly guideline PA time was at least 150 min. The classification was based on the MET values from the 6 s epochs as well as the 1 min and 6 min EMA MET values.

Participants were weighted by the sample size in age groups for men and women (20–34, 35–49 and 50–64 years). It was assumed that at the population level, there is equal distribution of individuals in age groups. All the analysis were done for the weighted data. Participants’ characteristics in CRF groups are presented as means and standard deviations for men and women. Differences between CRF groups were tested with independent samples *t*-test. 95% confidence intervals for the proportion of sufficiently physically active persons were calculated using the Clopper–Pearson exact method. The required, device-based, weekly guideline PA time was determined with the receiver operator characteristic (ROC) curve. The optimal cut-point was the point where the ROC curve was closest to the left-upper corner of the ROC space, and it denoted the minimum time required to achieve the VO_2_max value greater than or equal to 7.9 MET.

All statistical analyses were conducted using IBM SPSS statistics software (IBM SPSS Statistics for Windows, Version 27.0. Armonk, NY: IBM Corp., New York, NY, USA).

## Results

The number of participants in the low and adequate CRF groups is shown in table 1. The mean BMI of the low CRF group was higher than in the adequate CRF group. The proportion of the low CRF participants increased with age.

**Table 1:** Number of participants in CRF and age groups and mean (standard deviation) of the BMI

		Low CRF		Adequate CRF	
	Age group (years)	*N*	BMI (kg/m^2^)	*N*	BMI (kg/m^2^)
Men	20–34	3	40.5 (7.2)	114	25.7 (3.7)
	35–49	7	36.6 (7.5)	224	26.1 (3.7)
	50–64	35	31.0 (5.0)	275	26.5 (2.9)
Women	20–34	16	32.1 (4.3)	188	23.2 (3.1)
	35–49	49	34.4 (4.7)	288	23.8 (3.0)
	50–64	130	31.3 (4.3)	316	24.5 (2.9)

**Figure 1:**
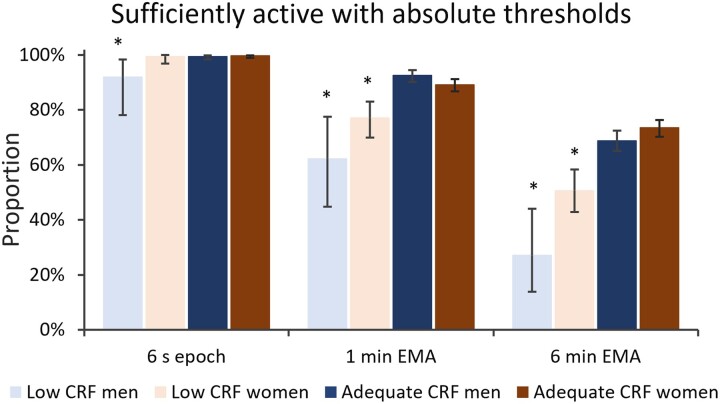
Proportions of the sufficiently physically active participants with absolute, 3.0 MET and 6.0 MET thresholds. The sufficiently active participants accumulated at least 150 min a week. The error bars denote for 95% confidence interval. The * denotes for significant (*P* < 0.05) difference between the sex-specific CRF groups

The proportions of participants adhering to the PA guidelines using the absolute MET-based thresholds are shown in figure 1. Except for the 6 s epoch and men, both the men and women in the adequate CRF group had higher adherence to PA guidelines than those in the low CRF group, whereas with the 6 s epoch almost all participants were sufficiently active. The device-based adherences were highest with the 6 s epoch and lowest with the 6 min EMA in each group. The weighted mean proportions of participants meeting the PA guidelines in the whole sample were 99.3% with the 6 s epoch, 88.9% with the 1 min EMA and 68.0% with the 6 min EMA.

**Figure 2:**
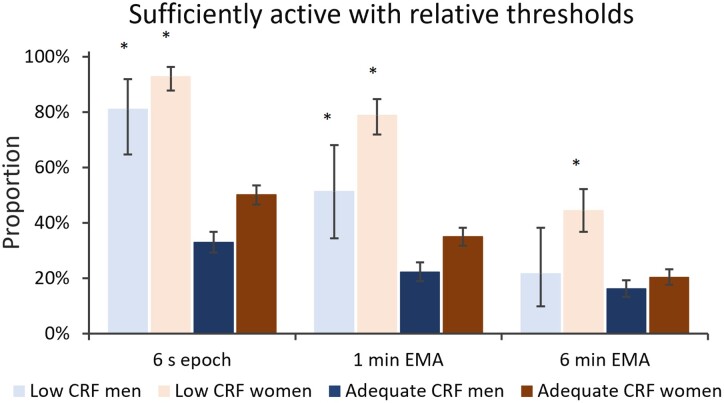
Proportions of the sufficiently physically active participants with relative 40% and 60% of the oxygen uptake reserve thresholds. The sufficiently active participants accumulated at least 150 min a week of guideline PA time. The error bars denote for 95% confidence interval. The * denotes for significant (*P* < 0.05) difference between the sex-specific CRF groups

The proportions of participants adhering to the present PA guidelines using the relative, individual CRF-based thresholds are shown in figure 2. The use of relative thresholds inverted the results and both men and women in the low CRF group had higher adherence to PA guidelines than those in the adequate CRF group except for the 6 min EMA, where the adherence of men was similar irrespective of the CRF group. The weighted mean proportions of participants meeting the PA guidelines in the whole sample were 46.6% with the 6 s epoch, 33.2% with the 1 min EMA and 20.4% with the 6 min EMA. Supplementary table S1 contains all values of figures 1 and 2.

Optimal cut-points for the accumulated weekly guideline PA time with absolute thresholds are shown in table 2. The cut-points define the required weekly device-based PA time to achieve the positive outcome, estimated VO_2_max ≥7.9 MET. The required time was highest for the 6 s epoch and lowest for the 6 min EMA with absolute thresholds. The area under the curve values regarding the CRF outcome were higher for men than women. The results for the relative thresholds are not shown because the low CRF group accumulated more guideline PA time than the adequate CRF group making the analysis of cut-points irrational.

**Table 2:** The cut-points (min/week) for device-based weekly guideline PA time to achieve at least 7.9 MET VO_2_max

Group	Epoch	Cut-point (min/week)	Sensitivity (%)	Specificity (%)	AUC (95% CI)
All	6 s epoch	491	67	65	0.708 (0.704–0.712)
	1 min EMA	289	63	69	0.702 (0.698–0.706)
	6 min EMA	156	69	58	0.679 (0.675–0.683)
Men	6 s epoch	491	67	65	0.782 (0.774–0.790)
	1 min EMA	278	66	65	0.776 (0.768–0.784)
	6 min EMA	156	69	58	0.750 (0.742–0.758)
Women	6 s epoch	518	63	69	0.680 (0.675–0.684)
	1 min EMA	293	63	66	0.676 (0.672–0.680)
	6 min EMA	208	62	68	0.672 (0.667–0.676)

AUC, the area under the receiver operator characteristics curve; 95% CI, 95% confidence intervals.

## Discussion

The proportion of adults meeting the current PA guidelines^4^ varied from marginal 20% to almost perfect 99% depending on the analysis parameters. The lowest adherence to the PA guidelines was attained when the analysis was based on the 6 min EMA and relative cut-points. The highest, virtually perfect adherence was attained with the 6 s epoch, absolute cut-points. These contradictory findings can be very confusing to health care professionals, who are not necessarily familiar with the device-based measurements of PA, and particularly what is the impact of analysis parameters on the results. The present results are in line with previous studies showing that the choice of the analyzing parameters is critical and confers a substantial effect on the prevalence of meeting the PA guidelines.^34^^,^^35^

As the total volume of recommended PA, at least 150 min per week, stems from studies employing self-reported MVPA time,^2^ it is expectable to have challenges with device-based methods.^35^ Therefore, self-reports can be the most practical method to measure the adherence to current recommendations and combination of different instruments (self-reports and devices) may provide a more holistic picture of PA.^12^ Furthermore, the self-reports can provide information on the specific type of activity, the context of the activity or location,^17^^,^^18^ e.g. leisure time MVPA is more advantageous than work time MVPA.^36^ However, after leaving the requirement of at least 10 min bout length, it is no more easy to estimate the weekly amount of PA at different intensities accumulating from bouts lasting few seconds. This change challenges not only device-based measurements but also the questionnaires.

The device-based methods provide more consistent results and capture all relevant movements or PA causing measurable acceleration irrespective of their duration.^13^^,^^37^ They have better sensitivity to measure the difference in risk reduction for a given amount of MVPA.^7^ The risk reduction for a similar amount of MVPA is about three times greater in magnitude when PA is assessed by accelerometry.^7^ Thus, future PA guideline development should consider the measurability of the recommendation.

Obviously, a very low-fit person perceives already 3.0 MET physical exertion very hard while a high-fit person perceives even 6.0 MET exertion light.^24^^,^^36^^,^^38^ Accordingly, using the absolute cut-points, the adequate CRF group had higher adherence to PA guidelines and accumulated more guideline PA time (figure 1). In contrast, the use of CRF-based relative cut-points inverted the results, and the low CRF group became paradoxically the most active one having higher adherence to PA guidelines (figure 2). This does not serve the purpose of general PA recommendations for the population. This bizarre result suggests that high fitness can protect from excess or strenuous PA. Thus, participants in the low CRF group get physically stressed already in their daily routines, whereas the participants with higher CRF engage in exercise on voluntary basis.^36^

In general, the use of absolute intensity cut-points will likely yield more reasonable results, since better fitness and higher PA are associated not only with higher adherence to the guidelines but also maintain the established associations with health outcomes.^24^^,^^38^ The use of an individually determined relative intensity, in turn, may be more appropriate for individual exercise prescriptions in PA counseling. In PA counseling, the appropriate PA intensity needs to be tailored individually, while the goal is to find solutions to behavioral changes that are easy to adopt and sustain.^38^ However, in the population-based samples and interventions, the use of relative intensity cut-points is likely problematic, when the varying intensity levels will have an impact on the amount of measured MVPA time in a confusing way.^24^ The low VO_2_max is also associated with higher body mass and BMI.^39^ Thus, changes in body composition during interventions would have an effect on the VO_2_max and individual intensity thresholds.

Altogether, we argue that physical fitness is primarily more important for health than PA. However, from the perspective of public health policy, it would not be sensible to encourage individuals to become fit.^40^ Instead individuals should be recommended to increase their activity to improve their fitness.^40^ Increasing PA at the population level will increase the overall fitness of the population and yield health benefits within the population.

By definition, the 1 min EMA and 6 min EMA processing smooth the PA data collected in the 6 s epochs. These processing methods remove short, sporadic activities and typically accumulate less total daily PA time than the 6 s epochs.^24^ For activities performed at steady intensity, like jogging, smoothing does not change the results. For an intermittent high-intensity activity, like ball games, the smoothed 1 min EMA and 6 min EMA can accumulate more PA time but at a lower intensity. The 6 s epoch is likely a better estimator to actual bodily movements produced by muscles whereas the smoothed data reflect better the time course of the metabolic and endocrine responses to muscle activity.^32^^,^^33^ However, it is possible that neither the 6 s epoch, 1 min EMA, nor 6 min EMA alone is better than the others, but they just reflect different PA patterns and thus need different requirements for the accumulated time.

In this cross-sectional study, the optimal cut-point between the CRF groups was about two to three times higher with the 6 s epoch than with the 6 min EMA for both outcome types. The same fitness output was achieved with either 491 min, 289 min or 156 min weekly guideline PA time. The closest match between the optimal cut-points for the device-based weekly guideline PA time and the 150 min limit of the updated guideline was achieved with the 6 min EMA. Also, the differences between men and women are noteworthy and warrant further evaluation. However, it might not be possible to harmonize the outputs of different measurement methods or choose appropriate analysis parameters in a reasonable and consistent way. Various PA measurement methods have different sensitivities to a broad range of positive health outcomes.^2^

## Conclusion

The recommendation of the weekly volume stems from the subjective self-reports while the device-based methods are sensitive to analysis parameters. However, both the subjective self-reports and the device-based analysis are challenged by the recent change in PA recommendations, especially leaving the 10 min minimum length of PA. Although the relative intensity cut-points are more feasible for individual PA counseling, the absolute MET-based cut-points provide a more appropriate viewpoint to assess the adherence in population-based samples and interventions. In the present study, the results analyzed with 6 min EMA resembles the targets set by PA guidelines. Furthermore, future PA guideline development should consider the measurability of the recommendation.

## Supplementary data

Supplementary data are available at *EURPUB* online.

## Supplementary Material

ckac078_Supplementary_Data

## References

[1] Mandsager K , Harb S, Cremer P, et al Association of cardiorespiratory fitness with long-term mortality among adults undergoing exercise treadmill testing. JAMA Netw Open 2018;1:e183605. 30646252 10.1001/jamanetworkopen.2018.3605 PMC6324439

[2] DiPietro L , Buchner DM, Marquez DX, et al New scientific basis for the 2018 U.S. Physical Activity Guidelines. J Sport Health Sci 2019;8:197–200. 31193291 10.1016/j.jshs.2019.03.007 PMC6525104

[3] Bull FC , Al-Ansari SS, Biddle S, et al World Health Organization 2020 guidelines on physical activity and sedentary behaviour. Br J Sports Med 2020;54:1451–62. 33239350 10.1136/bjsports-2020-102955 PMC7719906

[4] Piercy KL , Troiano RP, Ballard RM, et al The physical activity guidelines for Americans. JAMA 2018;320:2020–8. 30418471 10.1001/jama.2018.14854 PMC9582631

[5] Saint-Maurice PF , Troiano RP, Matthews CE, et al Moderate-to-vigorous physical activity and all-cause mortality: do bouts matter? J Am Heart Assoc 2018;7:e007678. 29567764 10.1161/JAHA.117.007678 PMC5907548

[6] Sjöros T , Vähä-Ypyä H, Laine S, et al Both sedentary time and physical activity are associated with cardiometabolic health in overweight adults in a 1 month accelerometer measurement. Sci Rep 2020;10:1–11. 33239818 10.1038/s41598-020-77637-3 PMC7688927

[7] Ekelund U , Dalene KE, Tarp J, Lee IM. Physical activity and mortality: what is the dose response and how big is the effect? Br J Sports Med 2020;54:1125–6. 31964630 10.1136/bjsports-2019-101765

[8] Ekelund U , Steene-Johannessen J, Brown WJ, et al Does physical activity attenuate, or even eliminate, the detrimental association of sitting time with mortality? A harmonised meta-analysis of data from more than 1 million men and women. Lancet 2016;388:1302–10. 27475271 10.1016/S0140-6736(16)30370-1

[9] Lee IM , Shiroma EJ, Lobelo F, et al; Lancet Physical Activity Series Working Group. Effect of physical inactivity on major non-communicable diseases worldwide: an analysis of burden of disease and life expectancy. Lancet 2012;380:219–29. 22818936 10.1016/S0140-6736(12)61031-9 PMC3645500

[10] Kodama S , Saito K, Tanaka S, et al Cardiorespiratory fitness as a quantitative predictor of all-cause mortality and cardiovascular events in healthy men and women: a meta-analysis. JAMA 2009;301:2024–35. 19454641 10.1001/jama.2009.681

[11] Warren JM , Ekelund U, Besson H, et al; Experts Panel. Assessment of physical activity—a review of methodologies with reference to epidemiological research: a report of the exercise physiology section of the European Association of Cardiovascular Prevention and Rehabilitation. Eur J Cardiovasc Prev Rehabil 2010;17:127–39. 20215971 10.1097/HJR.0b013e32832ed875

[12] Sattler MC , Ainsworth BE, Andersen LB, et al Physical activity self-reports: past or future? Br J Sports Med 2021;55:889–91. 33536193 10.1136/bjsports-2020-103595 PMC8477753

[13] Dowd KP , Szeklicki R, Minett MA, et al A systematic literature review of reviews on techniques for physical activity measurement in adults: a DEDIPAC study. Int J Behav Nutr Phys Act 2018;15:15–33. 29422051 10.1186/s12966-017-0636-2 PMC5806271

[14] Hukkanen H , Husu P, Sievänen H, et al Aerobic physical activity assessed with accelerometer, diary, questionnaire, and interview in a Finnish population sample. Scand J Med Sci Sports 2018;28:2196–206. 29923623 10.1111/sms.13244

[15] Luzak A , Heier M, Thorand B, et al; for the KORA-Study Group. Physical activity levels, duration pattern and adherence to WHO recommendations in German adults. PLoS One 2017;12:e0172503. 28245253 10.1371/journal.pone.0172503 PMC5330478

[16] Strain T , Fitzsimons C, Kelly P, Mutrie N. The forgotten guidelines: cross-sectional analysis of participation in muscle strengthening and balance & co-ordination activities by adults and older adults in Scotland. BMC Public Health 2016;16:1108. 27769211 10.1186/s12889-016-3774-6 PMC5073826

[17] Haskell WL. Physical activity by self-report: a brief history and future issues. J Phys Act Health 2012;9:S5–10. 22287448 10.1123/jpah.9.s1.s5

[18] Troiano RP , Pettee Gabriel KK, Welk GJ, et al Reported physical activity and sedentary behavior: why do you ask? J Phys Act Health 2012;9:S68–75. 22287450 10.1123/jpah.9.s1.s68

[19] Garber CE , Blissmer B, Deschenes MR, et al; American College of Sports Medicine. Quantity and quality of exercise for developing and maintaining cardiorespiratory, musculoskeletal, and neuromotor fitness in apparently healthy adults: guidance for prescribing exercise. Med Sci Sports Exerc 2011;43:1334–59. 21694556 10.1249/MSS.0b013e318213fefb

[20] Siddique J , Aaby D, Montag SE, et al Individualized relative intensity physical activity accelerometer cut points. Med Sci Sports Exerc 2020;52:398–407. 31524826 10.1249/MSS.0000000000002153 PMC6962549

[21] Bittner V , Weiner DH, Yusuf S, et al Prediction of mortality and morbidity with a 6-minute walk test in patients with left ventricular dysfunction. JAMA 1993;270:1702–7. 8411500

[22] Rostagno C , Gensini GF. Six minute walk test: a simple and useful test to evaluate functional capacity in patients with heart failure. Intern Emerg Med 2008;3:205–12. 18299800 10.1007/s11739-008-0130-6

[23] Mänttäri A , Suni J, Sievänen H, et al Six‐minute walk test: a tool for predicting maximal aerobic power (VO_2_ max) in healthy adults. Clin Physiol Funct Imaging 2018;38:1038–45. 10.1111/cpf.12525 29851229

[24] Vähä-Ypyä H , Sievänen H, Husu P, et al Intensity paradox—low-fit people are physically most active in terms of their fitness. Sensors 2021;21:2063. 33804220 10.3390/s21062063 PMC8002087

[25] Edwardson CL , Gorely T. Epoch length and its effect on physical activity intensity. Med Sci Sports Exerc 2010;42:928–34. 19996997 10.1249/MSS.0b013e3181c301f5

[26] Orme M , Wijndaele K, Sharp SJ, et al Combined influence of epoch length, cut-point and bout duration on accelerometry-derived physical activity. Int J Behav Nutr Phys Act 2014;11:34. 24612726 10.1186/1479-5868-11-34 PMC4008000

[27] Banda JA , Haydel KF, Davila T, et al Effects of varying epoch lengths, wear time algorithms, and activity cut-points on estimates of child sedentary behavior and physical activity from accelerometer data. PLoS One 2016;11:e0150534. 26938240 10.1371/journal.pone.0150534 PMC4777377

[28] Husu P , Tokola K, Vähä-Ypyä H, et al Physical activity, sedentary behavior, and time in bed among finnish adults measured 24/7 by triaxial accelerometry. J Meas Phys Behav 2021;4:163–73.

[29] Vähä-Ypyä H , Vasankari T, Husu P, et al A universal, accurate intensity‐based classification of different physical activities using raw data of accelerometer. Clin Physiol Funct Imaging 2015;35:64–70. 24393233 10.1111/cpf.12127

[30] Vähä-Ypyä H , Vasankari T, Husu P, et al Validation of cut-points for evaluating the intensity of physical activity with accelerometry-based mean amplitude deviation (MAD). PLoS One 2015;10:e0134813. 26292225 10.1371/journal.pone.0134813 PMC4546343

[31] Vähä-Ypyä H , Husu P, Suni J, et al Reliable recognition of lying, sitting, and standing with a hip‐worn accelerometer. Scand J Med Sci Sports 2018;28:1092–102. 29144567 10.1111/sms.13017

[32] Ball D. Metabolic and endocrine response to exercise: sympathoadrenal integration with skeletal muscle. J Endocrinol 2015;224:R79–95. 25431226 10.1530/JOE-14-0408

[33] Poole DC , Jones AM. Oxygen uptake kinetics. Compr Physiol 2011;2.2:933–96. 10.1002/cphy.c100072 23798293

[34] Matthews CE , Keadle SK, Berrigan D, et al Influence of accelerometer calibration approach on MVPA estimates for adults. Med Sci Sports Exerc 2018;50:2285–91. 29933344 10.1249/MSS.0000000000001691 PMC6193831

[35] Watson KB , Carlson SA, Carroll DD, Fulton JE. Comparison of accelerometer cut points to estimate physical activity in US adults. J Sports Sci 2014;32:660–9. 24188163 10.1080/02640414.2013.847278 PMC4589136

[36] Gupta N , Dencker-Larsen S, Rasmussen CL, et al The physical activity paradox revisited: a prospective study on compositional accelerometer data and long-term sickness absence. Int J Behav Nutr Phys Act 2020;17:1–9. 32690043 10.1186/s12966-020-00988-7 PMC7370435

[37] Skender S , Ose J, Chang-Claude J, et al Accelerometry and physical activity questionnaires—a systematic review. BMC Public Health 2016;16:515. 27306667 10.1186/s12889-016-3172-0 PMC4910242

[38] Kujala UM , Pietilä J, Myllymäki T, et al Physical activity: absolute intensity versus relative-to-fitness-level volumes. Med Sci Sports Exerc 2017;49:474–81. 27875497 10.1249/MSS.0000000000001134

[39] Santtila M , Pihlainen K, Koski H, et al Physical fitness in young men between 1975 and 2015 with a focus on the years 2005-2015. Med Sci Sports Exerc 2018;50:292–98. 28976492 10.1249/MSS.0000000000001436

[40] Blair SN , Cheng Y, Holder JS. Is physical activity or physical fitness more important in defining health benefits? Med Sci Sports Exerc 2001;33:S379–99. 11427763 10.1097/00005768-200106001-00007

