# Supplementary material for: How adherence to the updated physical activity guidelines should be assessed with accelerometer?
Source: Eur J Public Health. 2022 Aug 26;32(Suppl 1):i50–5. doi: 10.1093/eurpub/ckac078 (PMC9421411; doi:10.1093/eurpub/ckac078)
Supplement: ckac078_Supplementary_Data [file ckac078_supplementary_data.zip › _Supplement table S1.docx]

| Table S1. Proportions of the sufficiently physically active participants with absolute and relative cut-points. The low CRF group has VO_2_max less than 7.9 MET and adequate group at least 7.9 MET. The group ‘All’ contains participants from both CRF groups. | | | | | | | |
| --- | --- | --- | --- | --- | --- | --- | --- |
|  |  | Absolute cut-points  3.0 MET and 6.0 MET | | | Relative cut-points  40 % and 60 % of VO_2_R | | |
|  |  | 6 s epoch | 1 min EMA | 6 min EMA | 6 s epoch | 1 min EMA | 6 min EMA |
| Men |  |  |  |  |  |  |  |
|  | Low CRF | 92 % | 62 % | 27 % | 81 % | 51 % | 22 % |
|  | Adequate CRF | 99 % | 93 % | 69 % | 33 % | 22 % | 16 % |
|  | All | 99 % | 91 % | 66 % | 36 % | 24 % | 16 % |
| Women |  |  |  |  |  |  |  |
|  | Low CRF | 99 % | 77 % | 51 % | 93 % | 79 % | 44 % |
|  | Adequate CRF | 100 % | 89 % | 73 % | 50 % | 35 % | 20 % |
|  | All | 100 % | 87 % | 69 % | 59 % | 44 % | 25 % |
| Men and women | |  |  |  |  |  |  |
|  | Low CRF | 96 % | 70 % | 39 % | 87 % | 65 % | 33 % |
|  | Adequate CRF | 99 % | 91 % | 71 % | 42 % | 29 % | 18 % |
|  | All | 99 % | 89 % | 68 % | 47 % | 33 % | 20 % |
