# Supplementary figures and images for: How adherence to the updated physical activity guidelines should be assessed with accelerometer?
Source: Eur J Public Health. 2022 Aug 26;32(Suppl 1):i50–5. doi: 10.1093/eurpub/ckac078 (PMC9421411; doi:10.1093/eurpub/ckac078)

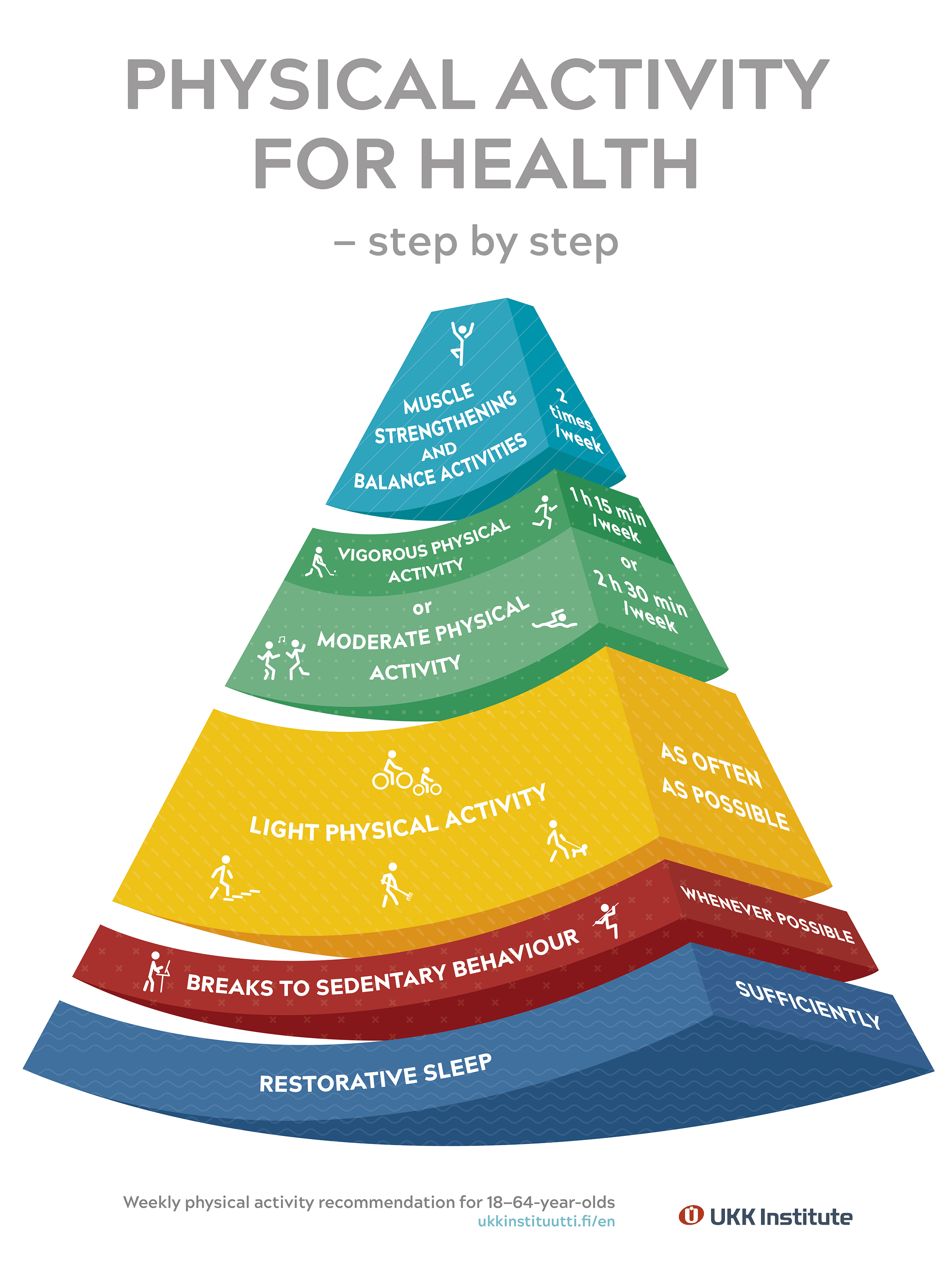

Supplement: ckac078_Supplementary_Data [file ckac078_supplementary_data.zip › FigureSupp1.tif]
